# Correction: Zhou, Z.-B. *et al*. Receptor-Mediated Vascular Smooth Muscle Migration Induced by LPA Involves p38 Mitogen-Activated Protein Kinase Pathway Activation. *Int. J. Mol. Sci.* 2009, *10*, 3194–3208

**DOI:** 10.3390/ijms10114953

**Published:** 2009-11-11

**Authors:** Zhi-Bin Zhou, Jian-Ping Niu, Zhi-Jun Zhang

**Affiliations:** 1 School of Clinical Medicine, Southeast University, Nanjing 21009, China; E-Mail: zhouzhibin74@163.com (Z.-B.Z.); 2 Department of Neurology, The second Hospital of Xiamen, Xiamen 361021, China

Due to personal reasons, I left the research group. In accordance with the regulations of the funding institution, Xiamen Health Administration, China, I hereby declare a withdrawal of my signature, Zhi-Jun Zhang, from this paper. Authors to whom correspondence should be addressed are therefore; E-Mails: Zhouzhibin74@163.com (Z.-B.Z.); nioujianp@yahoo.com.cn (J.-P.N.); Tel.: +86-25-83272024 (Z.-B.Z.); +86-519-6159635 (J.-P.N.); Fax: +86-25-83272011 (Z.-B.Z.); +86-519-6068389 (J.-P.N.).

We apologize for any inconvenience brought to the readers.

## References

[1] Zhou Z-B Niu J-P Zhang Z-J Receptor-Mediated Vascular Smooth Muscle Migration Induced by LPA Involves p38 Mitogen-Activated Protein Kinase Pathway Activation Int. J. Mol. Sci 2009 10 3194 3208 19742132 10.3390/ijms10073194 PMC2738919

